# Relationship Between Socioeconomic Status and Win-Win Values: Mediating Roles of Childhood Neglect and Self-Continuity

**DOI:** 10.3389/fpsyt.2022.882933

**Published:** 2022-05-13

**Authors:** Feng Zhang, Shan Zhang, Xu Gao

**Affiliations:** Institute of Psychology and Behavior, Henan University, Kaifeng, China

**Keywords:** socioeconomic status, win-win, childhood neglect, self-continuity, mediation effect

## Abstract

The family plays a key role on the development of children. One with low socioeconomic status was more likely to suffer childhood neglect, which might impact on development of self-continuity and win-win values. Using cross-sectional data from 489 participants, this study conducted a mediation model to examine the roles of childhood neglect and self-continuity between socioeconomic status and win-win values. Our results showed that childhood neglect and self-continuity fully mediated the effect of socioeconomic status on win-win values. Specifically, socioeconomic status might affect win-win values through three roles: the individual mediating role of childhood neglect, the individual mediating role of self-continuity, and the multiple mediation roles of childhood neglect and self-continuity.

## Introduction

According to previous research, a high rate of childhood neglect was observed worldwide (1). The experience of childhood trauma was extremely prevalent in the Asia-Pacific region, and neglect was the most common form of childhood trauma (2). Childhood neglect meant that a child’s basic needs were failed to be met by caregivers (3). Meanwhile, childhood neglect also included emotional neglect (failure to provide for the child’s basic emotional needs such as concern and love) and physical neglect (failure to provide for the child’s basic material needs such as food, safety, and medical health) (4). Approximately 28% of school-age children experienced emotional or physical neglect in China (5). A lot of studies have indicated that childhood neglect always brings negative effects to individuals. Most of the time, the effect of neglect lasts throughout people’s life (6). Moreover, the damage of neglect might cause permanent effects on mental health (7). For instance, some studies found that neglect could lead to loneliness, depression, and negative effects on social-emotion (8, 9). It is well known that the exposure to childhood neglect may increase the risk of several mental diseases. Childhood neglect may increase the risk of psychosis (10) and anxiety disorders (11). Childhood neglect may increase the risk to develop dysfunctional metacognitive beliefs (12) as well as the risk to engage in repetitive negative thinking such as rumination and worry (13). Broadly, childhood neglect limited the development of children and could alter self-perception, trust in others, perception of the world, and values (14).

Values were defined as wide motivational goals that guided one’s principles in life (15). Recently, the win-win values have been proposed, mainly reflecting situations where one actively considers and takes care of others to pursue personal interests (16, 17). Win-win was a combination of self-interest and mutual benefit in this globalized world. Childhood neglect might play a role of mediator between socioeconomic status (SES) and win-win values. First, socioeconomically disadvantaged children were more prone to be ignored. Childhood neglect was more common in low-income families than other traumas (18). Poverty was the most important predictor of child neglect (19). Children born in impoverished families were more likely to experience traumas (20). Second, values were developed during childhood and adolescence (21). Childhood neglect was associated with various adverse conditions in adolescence and adulthood, and it had a long-term effect on thinking, behavior, and relationships (22). Condly (23) thought that the impact of adverse events was that it caused the individual to re-evaluate one’s view of oneself and the world rather than the direct harm from these events. Therefore, childhood neglect might be of impact on win-win values.

Furthermore, SES might have a direct effect on win-win values. According to Bronfenbrenner ecosystem theory (24), the impact of the social environment on individuals was summed to a nested system. Among these, the impact of microsystems (including family, school, and peers) was highly significant for individuals. Although some mediating variables influenced the formation of values, families always played a key role on developing values (25, 26). In addition, young people often had similar values to their families (25). All the above evidence illustrated that the family was one of the most critical factors in the development of individuals’ values. Given that SES was an important aspect of family, which was defined as the social position or class according to an individual’s material and non-material social resources (27), we proposed that SES could affect win-win values.

The pathways from SES to win-win values, however, were complex and multifaceted. First, self-continuity might also play the role of mediator between SES and win-win values. Self-continuity was defined as the connection between one’s self in different temporal dimensions, consisting of a fundamental aspect of identity (28–32). According to the identity verification principle, individuals used feedback from their environment to determine the extent to which they achieved their ideal identity (33). In addition, SES played a central role in the construction of self-concept and temporal self (34, 35). As a family environment, SES could impact the individual’s self-continuity. Compared to individuals with high SES, those with low SES had poor self-continuity. Further, people would not be able to take responsibility for past actions or cooperate with others to secure future benefits if lacking self-continuity (36), making it difficult to develop win-win values. Second, SES also affected self-continuity through childhood neglect. Studies have shown that young people with low SES were more likely to experience trauma compared to the general population. Such trauma could have many negative consequences for future life (37). For example, childhood trauma could affect the development of the individual’s self-continuity, causing a split between different periods of the self (38). Thus, childhood neglect and self-continuity might play multiple mediating effects between SES and win-win values.

The aim of this study is to investigate the mediating roles of childhood neglect and self-continuity in the effect of SES on win-win values via structural equation modeling (SEM). Specifically, the present study proposed the following hypotheses: H1. Childhood neglect mediated the effect of SES on win-win values; H2. Self-continuity mediated the effect of SES on win-win values; H3. Childhood neglect and self-continuity played multiple mediating roles between SES and win-win values.

## Materials and Methods

### Participants

Participants were recruited from three universities by cluster random sampling in Henan province in China. A total of 575 questionnaires were distributed, and all participants completed the questionnaire in the classroom. After excluding invalid questionnaires (e.g., missing values, extreme responses, and outliers), data of 489 participants (112 males, 377 females) remained. Their ages ranged from 17 to 26 years (*M* = 20.72, *SD* = 1.43). This study was approved by the Research Ethics Committee of the Faculty of Education of Henan University. The participants provided their written informed consent to participate in this study.

### Measures

#### Socioeconomic Status Questionnaire

Three categories of socioeconomic status indicators in our measure were used: Parental education level (i.e., primary school or below; junior middle school; high school graduation; college education; or graduate-level education), parental occupation (i.e., agricultural laborer, unskilled worker, or unemployed people; manual worker, self-employed person, or skilled worker; ordinary manager, or junior professional technician; middle manager, or intermediate professional technician; or senior manager, or senior professional technician), and gross monthly family income (CNY) (i.e., less than 2,001; 2,001–3,000; 3,001–4,000; 4,001–5,000; 5,001–6,000; 6,001–7,000; 7,001–8,000; 8,001–9,000; 9,001–10,000; 10,001–11,000; 11,001–12,000; or more than 12,000).

We calculated a composite measure of the total socioeconomic class scores by summing the standard *Z*-scores of parental education level, parental occupation, and gross monthly family income (39–41). Higher scores meant higher SES.

#### Childhood Neglect Scale

Childhood neglect scale was a brief (10-item) self-report version of the neglect dimension extracted from the childhood trauma questionnaire (CTQ-SF) compiled by Fink and Bernstein (42), and Chinese version was revised by Fu et al. (43).

Childhood neglect scale included two dimensions: Emotional neglect and physical neglect (sample items: “I didn’t have enough to eat,” “I had to wear dirty clothes”). Participants scored each item on a 5-point Likert-type scale (1 = never true, 5 = very often true). The total scores per subscale ranged from 5-25, with the total scores ranging from 10 to 50. The Cronbach’s alpha coefficient of the childhood neglect scale in the current sample was 0.857.

#### Self-Continuity Questionnaire

The self-continuity questionnaire (44) consisted of an eight items (four personal-continuity items and four temporal-continuity items, e.g., “I feel connected with my past,” “The past and present flow seamlessly together”), and it measured relatively concrete perceptions of continuity between one’s past and present (44), using a 7-point Likert-type scale (1 = strongly disagree, 7 = strongly agree). Participants indicated how they felt about the relationship between their past and present selves (45). The Cronbach’s alpha coefficient of the questionnaire was 0.866 in this study.

#### Win-Win Scale

Participants completed the win-win scale (17) to assess their win-win values. It consisted of five dimensions such as integrity, advancement, altruism, harmoniousness, and coordination. It was comprised of 16 items (e.g., “I think honesty is the basis of win-win,” “I often think from the perspective of others,” “I often discuss problems with others”), and assessed with a five-point Likert-type scale (1 = completely disagree, 5 = completely agree). The Cronbach’s alpha coefficient of the win-win scale was 0.892 in the present study.

### Statistical Analysis

All statistical analyses were performed using SPSS 22.0 and Mplus 7.4. First, Harman’s one-factor test was performed (46) to test the common method bias of this study. Then, descriptive statistics were reported as mean and standard deviation. And the correlations coefficients among all variables were obtained. Next, our hypothetical mediation model was tested using structural equation modeling (SEM). Goodness of fit indices for SEM were as follows: ratio of Chi-square to the degree of freedom (χ^2^/*df*), root mean square error of approximation (RMSEA), comparative fit index (CFI), Tucker-Lewis index (TLI), and standardized root mean square residual (SRMR). In general, χ^2^/*df* should not exceed 3, RMSEA should be smaller than 0.08, CFI and TLI should be higher than 0.90, and SRMR should be smaller than 0.05 (47). Last, Mplus 7.4 was used to examine the indirect effect in the mediation model. 95% bias-corrected bootstrap confidence intervals (CI) were calculated using bootstrap methods (5,000 bootstrap samples) (48).

## Results

### Test of Common Method Bias

In the study, Harman’s one-factor test was employed to test for common method bias (46). All items were included in the factor analysis, and the result indicated that the first common factor explained 17.53% of the total variance, which was below 40%. Therefore, common method bias was not serious in our study.

### Descriptive Statistics and Correlation Analysis

The descriptive statistics and bivariate correlation results were shown in Table 1. SES had significantly positive correlations with self-continuity and win-win values. Both emotional and physical neglect had significantly negative correlations with SES, self-continuity, and win-win values. Win-win values had a significantly positive correlation with self-continuity.

**TABLE 1:** The Mean, *M* standard deviation (*SD*), and Pearson’s correlation coefficient.

Variables	*M* (*SD*)	1	2	3	4	5	6
1 Age	20.72(1.432)	–					
2 SES	0.002(1.829)	–0.074	–				
3 Childhood neglect	16.297(5.094)	–0.002	−0.121**	–			
4 Emotion neglect	8.875(3.357)	0.012	−0.101*	0.920**	–		
5 Physical neglect	7.422(2.400)	–0.020	−0.116*	0.836**	0.553**	–	
6 Self-continuity	4.847(0.870)	0.142**	0.147**	−0.250**	−0.209**	−0.238**	–
7 Win-win values	62.057(7.722)	0.040	0.108*	−0.399**	−0.345**	−0.364**	0.288**

*p < 0.05.

**p < 0.01.

### Examination of Multiple Mediation Model

In our structural equation model, gender and age were controlled as covariates. Before testing the mediation model, we conducted a structural equation modeling test on the relationship between SES and win-win values. The results showed that SES significantly predicted win-win values (β = 0.108, *t* = 2.391, *p* = 0.017, *R*^2^ = 0.012).

**FIGURE 1:**
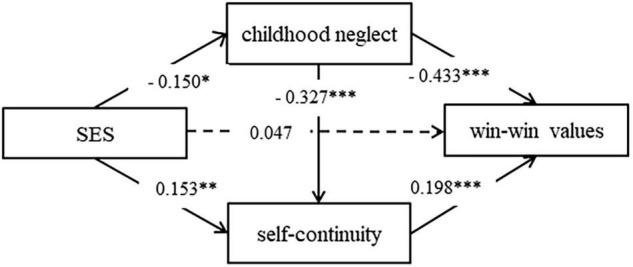
Path diagram of the mediation model. *p < 0.05, **p < 0.01, ***p < 0.001; dotted lines indicate the paths are not significant.

Then, we carried out a test of the mediation model. This model produced appropriate fit indices (χ^2^/*df* = 1.893, *p* < 0.001, RMSEA = 0.043, CFI = 0.967, TLI = 0.953, SRMR = 0.034). Figure 1 showed that all other path coefficients were significant in this model (*p*s < 0.05) except for the direct path from SES to win-win values.

The confidence intervals for the mediating effect did not include 0, indicating significant mediation effects. And the confidence interval for SES effect on win-win values included 0, which indicated that the direct effect was not significant (see Table 2). Complete mediation was present when the total and indirect effects were significant, while the direct effects were non-significant (49). As a result, the multiple mediation effects of childhood neglect and self-continuity between SES and win-win values were statistically significant.

**TABLE 2:** Mediation effect analysis and 95% confidence interval.

	Structural path	Effect	Ratio	95% CI	
				LL	UL
Direct effect	SES → win-win values	0.065		−0.075	0.205
Mediating effect	SES → childhood neglect → win-win values	0.090	61.6%	0.025	0.188
	SES → self-continuity → win-win values	0.042	28.8%	0.010	0.100
	SES → childhood neglect → self-continuity → win-win values	0.014	9.6%	0.004	0.037
Total indirect effect		0.146	100%	0.067	0.264

## Discussion

Childhood is the key and fragile stage of an individual’s life. Childhood neglect is at least as damaging as other traumas in the long term (50). Our study indicated that childhood neglect had significantly negative correlations with SES, win-win values, and self-continuity. Previous research found that families with low SES reported a high level of adverse events (51, 52). This included not only neglect from parents in a family, peers, and teachers in school, but also surroundings insecurity and other potential threats. These factors damaged children’s personality structure and adaptive functions. Living in an adverse family and social environment during childhood led to poor physical and mental problems, such as malnutrition and domestic violence (53). These problems hampered the development of cognition, psychology, and behavior (53), and may increase mortality and morbidity (54, 55). The results of the above studies might explain why childhood neglect was significantly negatively related to these research variables such as SES, win-win values, and self-continuity.

The present study further examined the mediation effect of childhood neglect between SES and win-win values, and the results showed that childhood neglect played a fully mediating role. It confirmed our first hypothesis (H1). First, chronic poverty was a significant risk factor for child neglect (56). Low SES was more strongly associated with neglect than other forms of childhood trauma (57, 58) and was also one of the most common risk factors in those experiencing chronic neglect (59). Second, a basic definition of childhood neglect was the parent or caregiver’s failure to meet children’s basic needs. Childhood neglect was often manifested in inadequate supervision and lack of concern for children’s well-being. Parents who were neglectful might provide the least cognitive enrichment (60). Third, parents were the predominant unit of socialization for children. Children might internalize and practice the values expressed in their parents’ behaviors. According to the above considerations, children with low SES lacked both rich cognitive stimulation and positive emotional connection with parents, which promoted maladaptive behavior and poor cognition. This situation might influence their values (14, 23), and it was subsequently difficult for them to build win-win values.

We found that self-continuity played a fully mediating role between SES and win-win values. The result confirmed our second hypothesis (H2). Additionally, our study revealed that childhood neglect and self-continuity played multiple mediating roles between SES and win-win values. The result confirmed our third hypothesis (H3). People from disadvantaged environments (e.g., low SES) were more likely to have experienced trauma (e.g., childhood neglect). Trauma-exposed people tended to experience a wide range of negative outcomes (e.g., low self-continuity) (37). Low self-continuity was associated with high social loneliness (61) and a mean-level decrease in agreeableness (62). It was very hard for people with low levels of self-continuity to develop win-win values. Therefore, lower SES individuals had lower win-win values in our study.

## Limitations

There are some limitations in this study. Our data collection and study design were cross-sectional. We cannot obtain causal effect among these variables, so causal interpretation should be cautious here. Moreover, in the present research, we focused solely on the mediating roles of childhood neglect and self-continuity. Future research could investigate other mediator or moderator variables to explore the influence adverse childhood experiences on the relationship between SES and win-win values in depth. Finally, we did not explore the differences between individuals who had suffered other childhood adversities (e.g., childhood abuse) and individuals who suffered childhood neglect. This issue should be explored in future studies.

## Conclusion

We concluded that socioeconomic status might influence win-win values by childhood neglect and self-continuity. Childhood neglect and self-continuity played multiple mediating roles between SES and win-win values.

Our study shed light on the mediating roles of childhood neglect and self-continuity between SES and win-win values, and thus confirmed the indirect mechanisms of SES effect on win-win values. First, low SES affected an individual’s experiences that brought childhood neglect, and indirectly affected an individual’s values. Second, low SES individuals who suffered physical and emotional neglect would be difficult to develop high self-continuity, and so their win-win values might be impacted. These results extended previous studies between SES and values.

At the same time, our results also suggested that low SES remained a significant risk factor for individual development. It was also prone to cause a series of subsequent problems of development. These problems would influence self-continuity and win-win values. Furthermore, values were meaningful predictors of mental health (63), we could increase self-continuity by reducing childhood neglect in order to develop win-win values. As a caregiver, parents could change their behaviors to reduce childhood adverse events. Thus, we should focus on the healthy development of childhood to lay a good foundation for the development of lifespan. In addition, our findings have clinical implication for the prevention of childhood neglect, and may be used for psychological interventions to form win-win values and construct higher self-continuity. When conducting psychological interventions, clinical counselors need to pay more attention to individuals with low SES in order to prevent childhood neglect.

## Data Availability Statement

The raw data supporting the conclusions of this article will be made available by the authors, without undue reservation.

## Ethics Statement

The studies involving human participants were reviewed and approved by Research Ethics Committee of the Faculty of Education of Henan University. The participants provided their written informed consent to participate in this study.

## Author Contributions

FZ and SZ contributed to conception and design of the study. XG performed the statistical analysis. SZ wrote the manuscript. All authors contributed to manuscript revision, read, and approved the submitted version.

## Conflict of Interest

The authors declare that the research was conducted in the absence of any commercial or financial relationships that could be construed as a potential conflict of interest.

## Publisher’s Note

All claims expressed in this article are solely those of the authors and do not necessarily represent those of their affiliated organizations, or those of the publisher, the editors and the reviewers. Any product that may be evaluated in this article, or claim that may be made by its manufacturer, is not guaranteed or endorsed by the publisher.

## References

[1] Bland VJ Lambie I Best C. Does childhood neglect contribute to violent behavior in adulthood? A review of possible links. *Clin Psychol Rev.* (2018) 60:126–35. 10.1016/j.cpr.2018.02.001 29454475

[2] Fulu E Miedema S Roselli T McCook S Chan KL Haardörfer R Pathways between childhood trauma, intimate partner violence, and harsh parenting: findings from the UN multi-country study on men and violence in Asia and the Pacific. *Lancet Glob Health.* (2017) 5:e512–22. 10.1016/S2214-109X(17)30103-1 28395846

[3] U.S. Department of Health Human Services. *Child Maltreatment 2012.* Washington, DC: U.S. Department of Health Human Services (2013).

[4] Teicher MH Samson JA. Annual research review: enduring neurobiological effects of childhood abuse and neglect. *J Child Psychol Psychiatry.* (2016) 57:241–66. 10.1111/jcpp.12507 26831814 PMC4760853

[5] UNICEF. *Measuring and Monitoring Child Protection Systems: Proposed Core Indicators for the East Asia and Pacific Region.* Bangkok: UNICEF EAPRO (2012).

[6] Ayhan AB Beyazit U. The associations between loneliness and self-esteem in children and neglectful behaviors of their parents. *Child Indic Res.* (2021) 14:1863–79. 10.1007/s12187-021-09818-z

[7] Nemeroff CB. Paradise lost: the neurobiological and clinical consequences of child abuse and neglect. *Neuron.* (2016) 89:892–909. 10.1016/j.neuron.2016.01.019 26938439

[8] Leeb RT Lewis T Zolotor AJ. A review of physical and mental health consequences of child abuse and neglect and implications for practice. *Am J Lifestyle Med.* (2011) 5:454–68. 10.1016/j.jflm.2020.101930 32452446

[9] Norman RE Byambaa M De R Butchart A Scott J Vos T. The long-term health consequences of child physical abuse, emotional abuse, and neglect: a systematic review and meta-analysis. *PLoS Med.* (2012) 9:e1001349. 10.1371/journal.pmed.1001349 23209385 PMC3507962

[10] Mansueto G Faravelli C. Stressful life events and psychosis: gender differences. *Stress Health.* (2022) 38:19–30. 10.1002/smi.3067 33973342

[11] Sperry DM Widom CS. Child abuse and neglect, social support, and psychopathology in adulthood: a prospective investigation. *Child Abuse Negl.* (2013) 37:415–25. 10.1016/j.chiabu.2013.02.006 23562083 PMC3672352

[12] Mansueto G Caselli G Ruggiero GM Sassaroli S. Metacognitive beliefs and childhood adversities: an overview of the literature. *Psychol Health Med.* (2019) 24:542–50. 10.1080/13548506.2018.1550258 30463429

[13] Mansueto G Cavallo C Palmieri S Ruggiero GM Sassaroli S Caselli G. Adverse childhood experiences and repetitive negative thinking in adulthood: a systematic review. *Clin Psychol Psychother.* (2021) 28:557–68. 10.1002/cpp.2590 33861493

[14] Dye H. The impact and long-term effects of childhood trauma. *J Hum Behav Soc Environ.* (2018) 28:381–92. 10.1080/10911359.2018.1435328

[15] Schwartz SH Cieciuch J Vecchione M Davidov E Fischer R Beierlein C Refining the theory of basic individual values. *J Pers Soc Psychol.* (2012) 103:663–88. 10.1037/a0029393 22823292

[16] Zhang F Zhang S. The structure exploration of the public views of co-win. *Commun Psychol Res (China).* (2020) 2:113–24.

[17] Zhang S Zang X Zhang F. Development and validation of the win-win scale. *Front Psychol.* (2021) 12:657015. 10.3389/fpsyg.2021.657015 34093348 PMC8175639

[18] Mawson A Gaysina D. Childhood socio-economic position and affective symptoms in adulthood: the role of neglect. *J Affect Disord.* (2021) 286:267–74. 10.1016/j.jad.2021.03.007 33752041

[19] Jonson-Reid M Drake B Zhou P. Neglect subtypes, race, and poverty: individual, family, and service characteristics. *Child Maltreat.* (2013) 18:30–41. 10.1177/1077559512462452 23109353 PMC3600388

[20] Paxton KC Robinson WL Shah S Schoeny ME. Psychological distress for African-American adolescent males: exposure to community violence and social support as factors. *Child Psychiatry Hum Dev.* (2004) 34:281–95. 10.1023/B:CHUD.0000020680.67029.4f 15039602

[21] Lewis-Smith I Pass L Reynolds S. How adolescents understand their values: a qualitative study. *Clin Child Psychol Psychiatry.* (2021) 26:231–42. 10.1177/1359104520964506 33070633 PMC7802049

[22] Meldrum RC Campion Young B Soor S Hay C Copp JE Trace M Are adverse childhood experiences associated with deficits in self-control? A test among two independent samples of youth. *Crim Justice Behav.* (2020) 47:166–86. 10.1177/0093854819879741

[23] Condly SJ. Resilience in children: a review of literature with implications for education. *Urban Educ.* (2006) 41:211–36. 10.1177/0042085906287902

[24] Bronfenbrenner U. *The Ecology of Human Development: Experiments by Nature and Design.* Cambridge, MA: Harvard university press (1979).

[25] Boehnke K Hadjar A Baier D. Parent-child value similarity: the role of zeitgeist. *J Marriage Fam.* (2007) 69:778–92. 10.1111/j.1741-3737.2007.00405.x

[26] Solomon S Knafo A. Value similarity in adolescent friendships. In: Rhodes TC editor. *Focus on Adolescent Behavior Research.* (Hauppauge, NY: Nova Science Publishers) (2007). p. 133–55.

[27] Zang X Jin K Zhang F. A difference of past self-evaluation between college students with low and high socioeconomic status: evidence from event-related potentials. *Front Psychol.* (2021) 12:629283. 10.3389/fpsyg.2021.629283 34054644 PMC8155721

[28] Breakwell GM. *Coping with Threatened Identities.* Hove: Psychology Press (2015).

[29] Erikson EH. *Identity: Youth and Crisis.* New York, NY: WW Norton & company (1968).

[30] Habermas T Köber C. Autobiographical reasoning in life narratives buffers the effect of biographical disruptions on the sense of self-continuity. *Memory.* (2015) 23:664–74. 10.1080/09658211.2014.920885 24912017

[31] Sedikides C Wildschut T Cheung WY Routledge C Hepper EG Arndt J Nostalgia fosters self-continuity: uncovering the mechanism (social connectedness) and consequence (eudaimonic well-being). *Emotion.* (2016) 16:524–39. 10.1037/emo0000136 26751632

[32] Sokol Y Serper M. Experimentally increasing self-continuity improves subjective well-being and protects against self-esteem deterioration from an ego-deflating task. *Identity.* (2019) 19:157–72. 10.1080/15283488.2019.1604350

[33] Reed A II Forehand MR Puntoni S Warlop L. Identity-based consumer behavior. *Int J Res Mark.* (2012) 29:310–21.

[34] Easterbrook MJ Kuppens T Manstead AS. Socioeconomic status and the structure of the self-concept. *Br J Soc Psychol.* (2020) 59:66–86. 10.1111/bjso.12334 31175690

[35] Antonoplis S Chen S. Time and class: how socioeconomic status shapes conceptions of the future self. *Self Identity.* (2021) 20:961–81. 10.1080/15298868.2020.1789730

[36] Becker M Vignoles VL Owe E Easterbrook MJ Brown R Smith PB Being oneself through time: bases of self-continuity across 55 cultures. *Self Identity.* (2018) 17:276–93. 10.1080/15298868.2017.1330222

[37] Craig JM. The potential mediating impact of future orientation on the ACE–crime relationship. *Youth Violence Juv Justice.* (2019) 17:111–28. 10.1177/1541204018756470

[38] Luyten P Campbell C Fonagy P. Borderline personality disorder, complex trauma, and problems with self and identity: a social-communicative approach. *J Pers.* (2020) 88:88–105. 10.1111/jopy.12483 31066053

[39] Bradley RH Corwyn RF. Socioeconomic status and child development. *Annu Rev Psychol.* (2002) 53:371–99. 11752490 10.1146/annurev.psych.53.100901.135233

[40] Cohen P Chen H Crawford TN Brook JS Gordon K. Personality disorders in early adolescence and the development of later substance use disorders in the general population. *Drug Alcohol Depend.* (2007) 88:S71–84. 10.1016/j.drugalcdep.2006.12.012 17227697 PMC2034357

[41] Zhang S Zang X Zhang S Zhang F. Social class priming effect on prosociality: evidence from explicit and implicit measures. *Int J Environ Res Public Health.* (2022) 19:3984. 10.3390/ijerph19073984 35409667 PMC8997543

[42] Fink L Bernstein D. *Childhood Trauma Questionnaire: A Retrospective Self-Report Manual.* San Antonio, TX: Harcourt Brace & Co (1998).

[43] Fu W Yao S Yu H Zhao X Li R Li Y Initial reliability and validity of childhood trauma questionnaire (CTQ-SF) applied in Chinese college students. *Chin J Clin Psychol.* (2005) 13:40–2.

[44] Sedikides C Wildschut T Routledge C Arndt J. Nostalgia counteracts self-discontinuity and restores self-continuity. *Eur J Soc Psychol.* (2015) 45:52–61. 10.1002/ejsp.2073

[45] Jiang T Chen Z Wang S Hou Y. Ostracism disrupts self-continuity. *Pers Soc Psychol Bull.* (2020) 47:1390–400. 10.1177/0146167220974496 33272111

[46] Podsakoff PM MacKenzie SB Lee JY Podsakoff NP. Common method biases in behavioral research: a critical review of the literature and recommended remedies. *J Appl Psychol.* (2003) 88:879–903. 10.1037/0021-9010.88.5.879 14516251

[47] Wu M. *Structural Equation Model: Operation and Application of the AMOS.* Chongqing: Chongqing University Press (China) (2009).

[48] Preacher KJ Hayes AF. SPSS and SAS procedures for estimating indirect effects in simple mediation models. *Behav Res Methods Instrum Comput.* (2004) 36:717–31. 10.3758/bf03206553 15641418

[49] Mtintsilana A Micklesfield LK Chorell E Olsson T Shivappa N Hebert JR Adiposity mediates the association between the dietary inflammatory index and markers of type 2 diabetes risk in middle-aged black South African women. *Nutrients.* (2019) 11:1246. 10.3390/nu11061246 31159253 PMC6628082

[50] Gilbert R Widom CS Browne K Fergusson D Webb E Janson S. Burden and consequences of child maltreatment in high-income countries. *Lancet.* (2009) 373:68–81. 10.1016/S0140-6736(08)61706-7 19056114

[51] Merrick MT Ford DC Ports KA Guinn AS. Prevalence of adverse childhood experiences from the 2011-2014 behavioral risk factor surveillance system in 23 states. *JAMA Pediatr.* (2018) 172:1038–44. 10.1001/jamapediatrics.2018.2537 30242348 PMC6248156

[52] Mock SE Arai SM. Childhood trauma and chronic illness in adulthood: mental health and socioeconomic status as explanatory factors and buffers. *Front Psychol.* (2011) 1:246. 10.3389/fpsyg.2010.00246 21833299 PMC3153850

[53] Yuan L. *Annual Report on Chinese Children’s Development.* Beijing: Social Sciences Academic Press (China) (2020).

[54] Cohen S Janicki-Deverts D Chen E Matthews KA. Childhood socioeconomic status and adult health. *Ann N Y Acad Sci.* (2010) 1186:37–55. 10.1111/j.1749-6632.2009.05334.x 20201867

[55] Shonkoff JP Boyce WT McEwen BS. Neuroscience, molecular biology, and the childhood roots of health disparities: building a new framework for health promotion and disease prevention. *JAMA.* (2009) 301:2252–9. 10.1001/jama.2009.754 19491187

[56] Slack KS Holl JL McDaniel M Yoo J Bolger K. Understanding the risks of child neglect: an exploration of poverty and parenting characteristics. *Child Maltreat.* (2004) 9:395–408. 10.1177/1077559504269193 15538038

[57] Drake B Pandey S. Understanding the relationship between neighborhood poverty and specific types of child maltreatment. *Child Abuse Negl.* (1996) 20:1003–18. 10.1016/0145-2134(96)00091-9 8958452

[58] Jones ED McCurdy K. The links between types of maltreatment and demographic characteristics of children. *Child Abuse Negl.* (1992) 16:201–15. 1559169 10.1016/0145-2134(92)90028-p

[59] Jones AS Logan-Greene P. Understanding and responding to chronic neglect: a mixed methods case record examination. *Child Youth Serv Rev.* (2016) 67:212–9.

[60] Font SA Maguire-Jack K. It’s not “Just poverty”: educational, social, and economic functioning among young adults exposed to childhood neglect, abuse, and poverty. *Child Abuse Negl.* (2020) 101:104356. 10.1016/j.chiabu.2020.104356 31931322 PMC7027312

[61] Lampraki C Jopp DS Spini D Morselli D. Social loneliness after divorce: time-dependent differential benefits of personality, multiple important group memberships, and self-continuity. *Gerontology.* (2019) 65:275–87. 10.1159/000494112 30605909

[62] Dunkel CS Worsley SK. Does identity continuity promote personality stability? *J Res Pers.* (2016) 65:11–5. 10.1016/j.jrp.2016.09.001

[63] Maercker A Zhang XC Gao Z Kochetkov Y Lu S Sang Z Personal value orientations as mediated predictors of mental health: a three-culture study of Chinese, Russian, and German university students. *Int J Clin Health Psychol.* (2015) 15:8–17. 10.1016/j.ijchp.2014.06.001 30487817 PMC6224790

